# Impact of the Thrombectomy Trials on the Management and Outcome of Large Vessel Stroke: Data From the Lyon Stroke Center

**DOI:** 10.3389/fneur.2018.00722

**Published:** 2018-08-28

**Authors:** Louis Viannay, Julie Haesebaert, Fannie Florin, Roberto Riva, Laura Mechtouff, Benjamin Gory, Elodie Ong, Paul-Emile Labeyrie, Laurent Derex, Marc Hermier, Leila Chamard, Lise-Prune Berner, Roxana Ameli, Yves Berthezène, Francis Turjman, Norbert Nighoghossian, Tae-Hee Cho

**Affiliations:** ^1^Department of Neuroradiology, Université Lyon 1, Hospices Civils de Lyon, Lyon, France; ^2^Health Information Department, HESPER EA7425, Université Lyon 1, Hospices Civils de Lyon, Lyon, France; ^3^Department of Stroke Medicine, Université Lyon 1, Hospices Civils de Lyon, Lyon, France; ^4^CREATIS, CNRS UMR 5220-INSERM U1206, INSA-Lyon, Hospices Civils de Lyon, Lyon, France

**Keywords:** acute ischemic stroke, large-vessel stroke, thrombectomy, endovascular procedures, clinical outcome, systems of care

## Abstract

**Introduction:** Randomized trials (RT) have recently validated the superiority of thrombectomy over standard medical care, including intravenous thrombolysis (IVT). However, data on their impact on routine clinical care remains scarce.

**Methods:** Using a prospective observational registry, we assessed: (1) the clinical and radiological characteristics of all consecutive patients treated with thrombectomy; (2) the outcome of all patients with M1 occlusion (treated with thrombectomy or IVT alone). Two periods were compared: before (2013–2014) and after (2015–2016) the publication of RT.

**Results:** Endovascular procedures significantly increased between the two periods (*N* = 82 vs. 314, *p* < 0.0001). In 2015–2016, patients were older (median [IQR]: 69 [57-80]; vs. 66 [53-74]; *p* = 0.008), had shorter door-to-clot times (69 [47-95]; vs. 110 [83-155]; *p* < 0.0001) resulting in a trend toward shorter delay from symptom onset to reperfusion (232 [185-300]; vs. 250 [200-339]; *p* = 0.1), with higher rates of reperfusion (71 vs. 48%; *p* = 0.0001). Conversely, no significant differences in baseline NIHSS scores, ASPECTS, delay to IVT or intracranial hemorrhage were found. In 2015–2016, patients with M1 occlusion were treated with thrombectomy more often than in 2013–2014 (87 vs. 32%, respectively; *p* < 0.0001), with a significant improvement in clinical outcome (shift analysis, lower modified Rankin scale scores: OR = 1.68; 95% CI: 1.10–2.57; *p* = 0.017).

**Conclusion:** Following the publication of RT, thrombectomy was rapidly implemented with significant improvements in intrahospital delay and reperfusion rates. Treatment with thrombectomy increased with better clinical outcomes in patients with M1 occlusion.

## Introduction

In 2013, the future of endovascular therapy (EVT) in acute ischemic stroke was uncertain, as three consecutive randomized trials failed to demonstrate the superiority of thrombectomy combined with intravenous thrombolysis (IVT) over IVT alone (1–3). Subsequent trials have since established the effectiveness of EVT for patients with large vessel occlusions who were suitably selected by cerebral and arterial imaging (4–11).

Preliminary reports from monocentric (12, 13) or multicentric (14, 15) studies indicate that EVT seems applicable in the “real” world of clinical practice, with similar results to those of controlled trials. Still, little data is available on how systems of care have started to adapt to this paradigm shift in acute stroke therapy. A single study recently reported on the increasing EVT case volumes across the Unites States since the publication of the positive trials (16). The extent to which EVT use has evolved after the pivotal trials and its impact on local practices need to be considered to plan further quality improvement efforts, both within comprehensive stroke centers (CSC, i.e., EVT-capable hospitals) and beyond.

**Figure 1:**
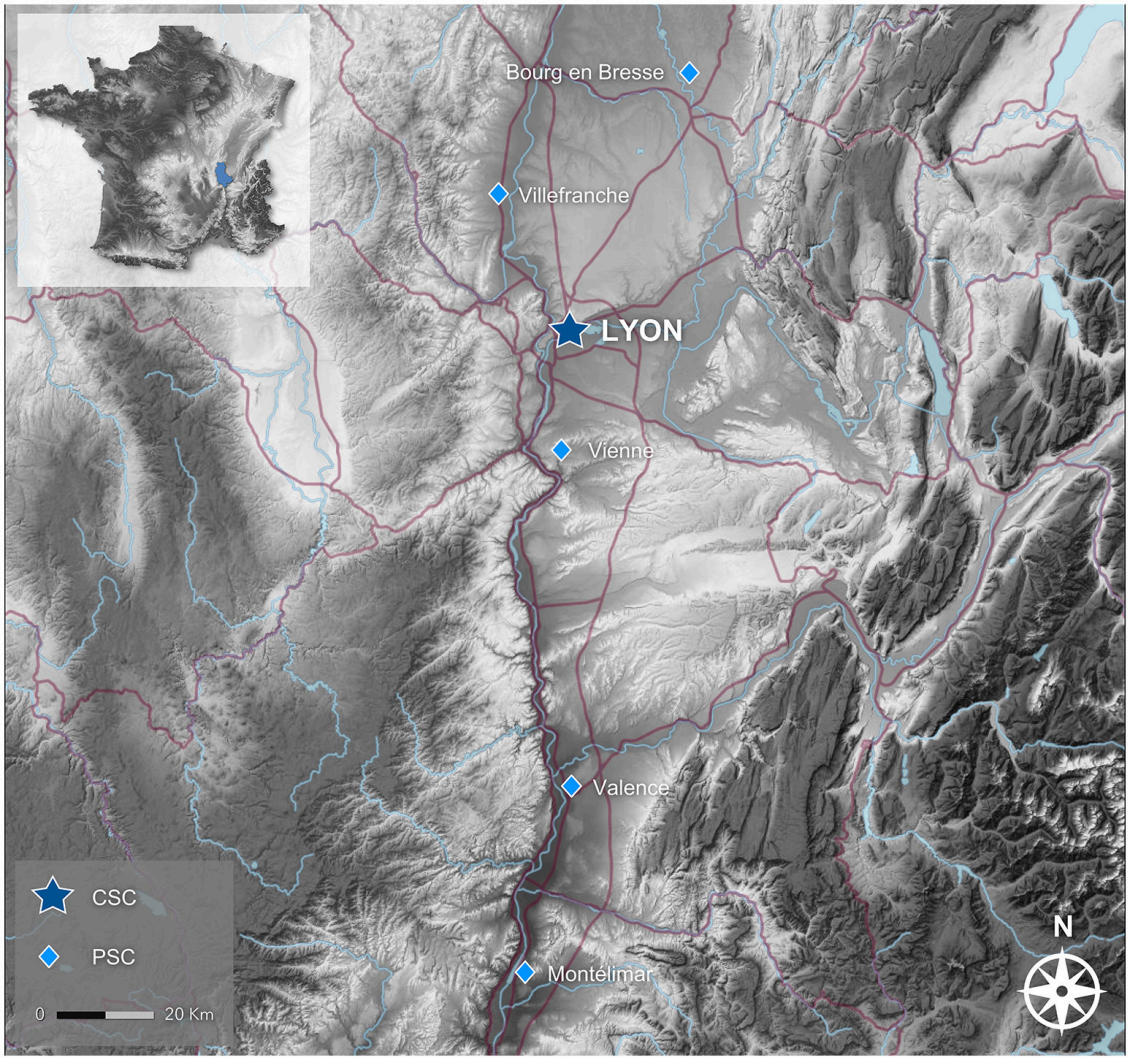
Stroke care network in the northern Rhône valley (inset, location within France; CSC, Comprehensive Stroke Center; PSC, Primary Stroke Center). The Lyon CSC is the only stroke unit in the Lyon urban area, and is the only referral center for thrombectomy for the PSCs showed in this map. Source: Institut national de l'information géographique et forestière (IGN).

Our institution is the only CSC serving the greater Lyon metropolitan area (population: 2.3 million), treating ~1,600 ischemic stroke patients each year (Figure 1). Prior to the publication of the first positive trial (4), EVT was not considered as standard care, and thus was not systematically considered for patients with proximal intracranial occlusions. Thereafter, local processes were modified to implement thrombectomy in all eligible patients referred to our CSC. Our objective was to assess the effects of this major shift in our reperfusion strategy by comparing two periods: before (January 1st 2013–December 31st 2014) and after (January 1st 2015–December 31st 2016) the publication of the first positive EVT trial. Specifically, we first compared the typology of all consecutive EVT cases (e.g., number of procedures, baseline clinical and radiological characteristics) to assess the development of EVT within our institution. Secondly, we compared the outcome of all patients with M1 occlusion who underwent a revascularization procedure (IVT and/or EVT).

## Methods

### Patients and treatment strategy

We identified all consecutive patients treated by IVT and/or EVT during a 4-year period, before (“PRE”: 2013–2014) and after (“POST”: 2015–2016) the publication of the first positive EVT trial (4). Data from these patients were collected within a regional emergency stroke network registry (RESUVAL), approved by the local ethics committee (Comité de Protection des Personnes Sud-Est II, registration E-2012-069). This observational study was carried out in accordance with the ethical standards of the Declaration of Helsinki. All patients gave informed consent.

The acute reperfusion strategy used in Lyon significantly evolved between these two periods. Before 2015, although our center was EVT-capable, thrombectomy was not considered standard care and thus was not systematically considered for patients with proximal occlusions. From 2015 onwards, local treatment protocols and workflows were adapted in order to integrate and make EVT available for all eligible patients. The target population was defined according to the recent trials (i.e., M1 occlusion, ischemic core estimated to be no more than a third of the MCA territory, EVT initiated within 6 h of onset) (17). No upper age-limit was enforced. Treatment decisions regarding both IVT and EVT in clinical situations not specifically addressed in randomized trials available during the study period (e.g., unknown time of onset, mild neurological symptoms, large ischemic core, distal and basilar artery occlusions) were left to the judgment of the treating medical team.

The following clinical parameters were recorded during routine patient care: age and gender; baseline National Institutes of Health Stroke Scale (NIHSS) score; delays between symptoms onset, initiation of IVT and/or EVT (defined by the opening of the stent retriever or aspiration of the clot, not groin puncture) and angiographic reperfusion; and the modified Rankin Scale (mRS) at 3 months.

### Imaging protocol

The first-line imaging method was MRI, including the following sequences: diffusion-weighted imaging (DWI), T2^*^-weighted imaging, Fluid-Attenuated-Inversion-Recovery (FLAIR), 3D-Time-of-Flight MR-angiography (MRA); perfusion-weighted imaging and cervical-vessels angiography were optional. If MRI was unavailable or contra-indicated, non-enhanced computed tomography (CT) followed by CT-angiography and CT-perfusion were performed. Baseline ischemic core size was assessed on DWI or CT using the Alberta Stroke Program Early CT Score (ASPECTS) for patients with stroke in the middle cerebral artery territory (18). Baseline arterial occlusion site was evaluated with MRA or CT-angiography.

The EVT technique was left to the discretion of the neuro-interventionists; all patients were treated using authorized stent retrievers and/or thrombo-aspiration devices. Angiographic reperfusion was defined by a Thrombolysis in Cerebral Infarction (TICI) score of 2b or 3 (19).

Hemorrhagic transformation was evaluated on a follow-up CT at ~24 h, using the European Cooperative Acute Stroke Study (ECASS2) criteria (20).

### Statistical analysis

Clinical and imaging variables were described as median and interquartile range (IQR) or proportions as appropriate. Two distinct analyses were performed.

Firstly, we compared the baseline characteristics and angiographic outcome of all patients treated with EVT, regardless of occlusion site, between the PRE and POST periods. Statistical significance for intergroup differences was assessed with the Wilcoxon rank-sum test for continuous variables, and Fisher exact test for categorical variables. The objective of this analysis was to assess any difference in EVT use, patient characteristics or technical quality endpoints (e.g., treatment delays and reperfusion rates) between the two periods.

Secondly, we assessed the clinical impact of the treatment strategy modifications between the two periods in patients with M1 occlusion (with or without internal carotid artery occlusion) who were directly admitted to our CSC, and subsequently treated by IVT and/or EVT. This distinct analysis thus included patients who only received IVT without thrombectomy (mostly during the PRE period). Patients transferred from another hospital (e.g., distant primary stroke centers) were not included to avoid uncontrolled biases. Indeed, data were not available for patients treated outside our institution (e.g., patients treated only by IVT in the PRE period). We conducted univariate and multivariate ordinal logistic regression to identify factors associated with 3-month mRS in all patients with M1 occlusions treated by either IVT alone or EVT with or without IVT. The following baseline patient characteristics were included: age, gender, NIHSS score, ASPECTS and the period (PRE vs. POST).

Statistical analyses were performed using SAS (version 9.4 Cary, NC: SAS Institute Inc.).

## Results

During the 4-year period (2013–2016), 5,480 patients with ischemic stroke were admitted in our CSC; 1,056 patients (19.3%) were treated with either IVT alone or EVT (associated or not with IVT). Among these, EVT was used in 396 patients (37.5%).

### Evolution of endovascular procedures

The number of endovascular procedures significantly increased between the two periods (*N* = 82 vs. 314, *p* < 0.0001). EVT thus represented 18.2% of all reperfusion procedures before 2015 (including patients treated with IVT alone), increasing to 51.8% in 2015–2016. The clinical and imaging characteristics of all patients treated with EVT during the study period are summarized in Table 1.

**Table 1:** Clinical and imaging characteristics of all patients treated with thrombectomy.

	**2013–2014 (*N* = 82)**	**2015–2016 (*N* = 314)**	***P***
Age	66 (53–74)	69 (57–80)	**0.008**
Women, *N* (%)	36 (44%)	142 (45%)	0.9
NIHSS	19 (12–21)	17 (11–21)	0.34
ASPECTS	8 (5–9)	8 (6–9)	0.81
IVT, *N* (%)	49 (59.8)	224 (71.3)	0.06
Patients transferred from another hospital, *N* (%)	24 (29.3)	122 (38.8)	0.12
**DELAYS**			
Onset to IVT	120 (105–179)	135 (112–170)	0.77
Door to IVT	49 (40–58)	44 (35–56)	0.3
Onset to clot	210 (185–276)	220 (167–289)	0.88
Door to clot	110 (83–155)	69 (47–95)	<**0.0001**
Onset to reperfusion^**^	250 (200–339)	232 (185–300)	0.1
**OCCLUSION SITE**			
Anterior circulation, *N* (%)	57 (69.5)	277 (88.2)	**0.0001**
Basilar occlusion, *N* (%)	25 (30.5)	37 (11.8)	
Reperfusion (TICI 2b-3), %	48	71	**0.0001**
Any hemorrhagic transformation, N (%)	29 (35.4)	101 (32.2)	0.6
Parenchymal hematoma type 2, N (%)	4 (4.9)	8 (2.5)	0.28

*In all Tables, values are expressed as median and interquartile range, unless otherwise indicated. IVT: intravenous thrombolysis. EVT: endovascular therapy. Bold values indicate P < 0.05*.

** *only in patients with angiographic reperfusion (TICI score 2b-3)*.

In 2015–2016, patients were older (median [IQR]: 69 [57-80]; vs. 66 [53-74]; *p* = 0.008), had a shorter intrahospital delay to EVT initiation (i.e., “door-to-clot” times: 69 [47-95]; vs. 110 [83-155]; *p* < 0.0001) resulting in a trend toward shorter delay from symptom onset to reperfusion (232 [185-300] vs. 250 [200-339]; *p* = 0.1), with higher rates of reperfusion (71 vs. 48%; *p* = 0.0001). The occlusion site significantly evolved between the two periods: in 2013–2014, basilar artery occlusions represented 30.5% of all endovascular procedures, compared to only 11.8% in 2015–2016 (*p* = 0.0001). Conversely, no significant differences in baseline NIHSS scores, ASPECTS, proportion of transferred patients, delay to IVT or intracranial hemorrhage were found.

### Clinical outcome of patients with M1 occlusions

**Figure 2:**
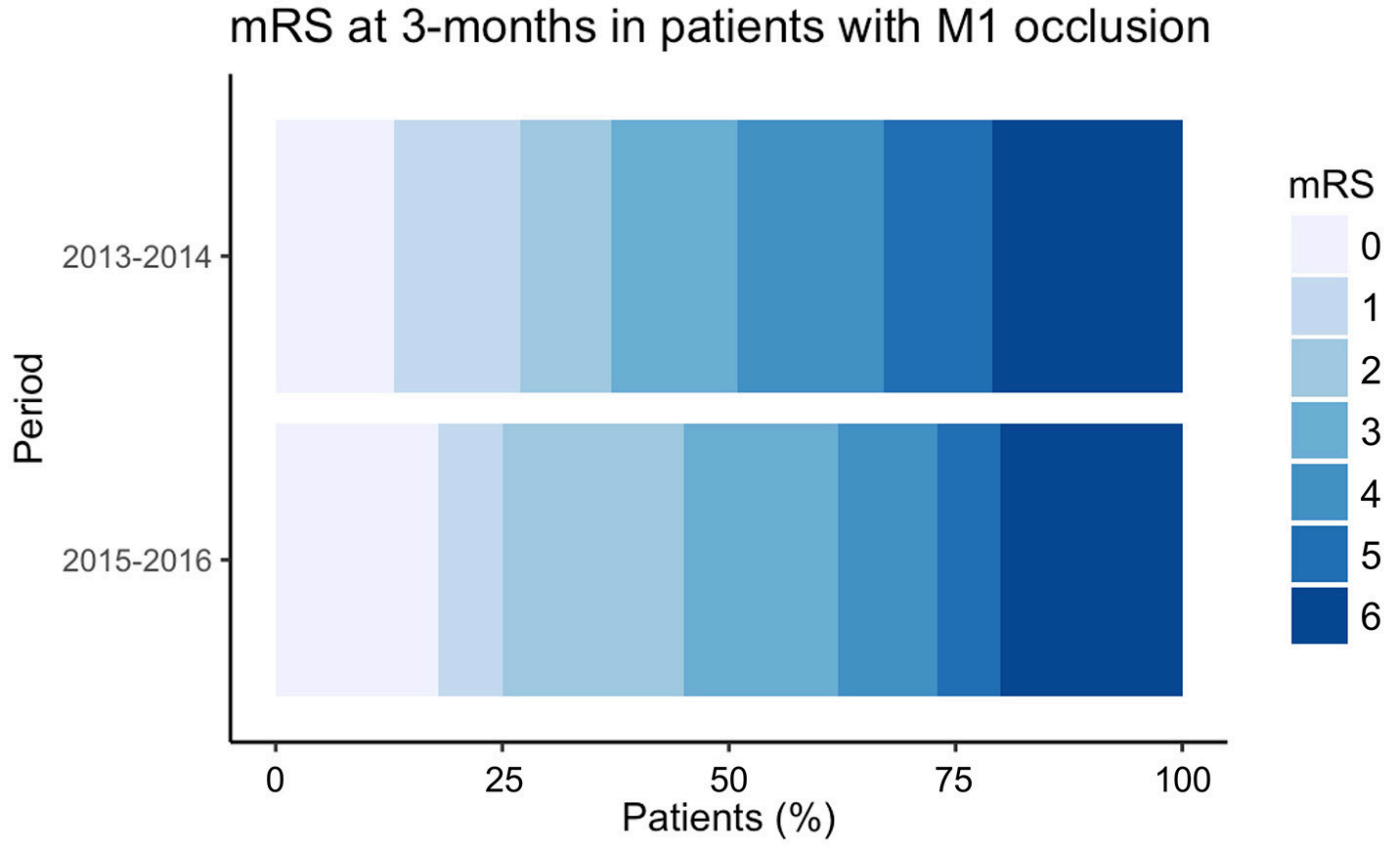
Distribution of the modified Rankin Scale at 3-month of patients admitted with a M1 occlusion before (2013–2014) and after (2015–2016) the publication of the positive randomized trials.

Patients with M1 occlusion represented 31% (*N* = 326) of all reperfusion procedures (IVT alone and EVT with or without IVT) during the study period. The clinical and imaging characteristics of patients with M1 occlusion are summarized in Table 2. Patients in the POST period were more often male and showed a trend for more severe neurological deficits (Table 2). The proportion of patients with M1 occlusion treated with EVT significantly increased from 2015 onwards (87 vs. 32%, in POST and PRE period, respectively; *p* < 0.0001). When adjusted for baseline variables (age, gender, NIHSS score and ASPECTS), an improved clinical outcome was observed after implementation of EVT (shift analysis, lower modified Rankin scale scores: OR = 1.68; 95% CI: 1.096–2.566; *p* = 0.017; Table 3 and Figure 2).

**Table 2:** Clinical and imaging characteristics of patients with M1 occlusion.

	**2013–2014**	**2015–2016**	***P***
	**(*N* = 151)**	**(*N* = 175)**	
Age	73.7 (62.9–83.0)	70.1 (59.3–83.3)	0.426
Women, *N* (%)	86 (56.9)	79 (45.1)	**0.033**
NIHSS	17 (12–21)	19 (14–22)	0.057
ASPECTS	8 (6–9)	7 (6–9)	0.635
IVT, *N* (%)	130 (86.1)	130 (74.3)	**0.008**
EVT, *N* (%)	48 (31.8)	152 (86.7)	<**0.0001**
**DELAYS**			
Onset to IVT	150 (116–200)	131 (108–172)	**0.019**
Door to IVT	50 (38–63)	42 (33–55)	**0.012**
Onset to clot	195 (163–230)	187 (154–227)	0.463
Door to clot	125 (98–140)	80 (66–105)	**0.002**
Onset to reperfusion^**^	223 (192–328)	203 (160–255)	0.463
Reperfusion (TICI 2b-3), %	41.7	71.7	**0.0001**
Any hemorrhagic transformation, *N* (%)	46 (30.8)	55 (32.0)	0.832
Parenchymal hematoma type 2, *N* (%)	8 (5.3)	7 (4.0)	0.61

** *only in patients with angiographic reperfusion (TICI score 2b-3). Bold values indicate P < 0.05*.

**Table 3:** Univariate and multivariate ordinal logistic regression for final mRS.

	**Crude OR**	**95%CI**	***P***	**Adjusted OR**	**95%CI**	***P***
Period (POST vs. PRE)	1.304	0.882–1.927	0.183	1.677	1.096–2.566	0.017
Gender	0.897	0.608–1.323	0.583	0.95	0.615–1.469	0.819
Age (for 1 year increase)	0.959	0.946–0.972	<0.0001	0.948	0.934–0.963	<0.0001
NIHSS (for 1 point increase)	0.893	0.863–0.925	<0.0001	0.916	0.881–0.953	<0.0001
ASPECTS (for 1 point increase)	1.237	1.121–1.366	<0.0001	1.289	1.151–1.442	<0.0001

## Discussion

In this longitudinal study, we evaluated the impact of the recent thrombectomy trials on the only CSC serving a large metropolitan area. Though previously EVT-capable, thrombectomy was not part of our standard care prior to the positive randomized trials. We have shown that a rapid deployment of thrombectomy is feasible in such a setting, with improved clinical outcomes for patients with M1 occlusion. An overhaul of acute treatment workflows made EVT available for all eligible patients admitted to our CSC.

A single study previously assessed the evolution of thrombectomy case volumes following the positive EVT trials, across 2,222 hospitals in the United States (16). An increase in thrombectomy was observed, especially in previously EVT-capable centers where case volumes nearly doubled from 2013 to 2016. In contrast, a ~4-fold increase in endovascular procedures occurred within 2 years in our center, with EVT accounting for over half of all acute revascularization procedures from 2015 onwards. This sharp increase is in part related to the centralized nature of the stroke care network in our metropolitan area, as evidenced by the proportion of transferred patients (~40% of all EVT cases in 2015–2016). Other urban centers with similar organizations (single CSC) may experience comparable rates of thrombectomy uptake.

The incidence of patients with large-vessel occlusions who are eligible for thrombectomy (admission within 5–6 h of symptoms onset, no large ischemic core) is estimated at 10–22 cases per 100,000 person-years (21, 22). Assuming these rates, ~220 to 500 EVT-eligible patients per year can be expected within our urban area, with even higher numbers if extended time window trials (10, 11) and our entire catchment area are considered (our CSC receive patients from as far as 150 km south in the Rhône valley; Figure 1). As elsewhere, we estimate that no more than 40–50% of locally eligible patients are currently treated with thrombectomy. Notwithstanding the required improvements in the prehospital phase, and the ongoing debate on the optimal access to thrombectomy (centralized “mothership” vs. “drip-and-ship” paradigms) (23, 24), substantial increases in staffing and imaging platforms will be required in many CSC.

Previous limited monocentric (*N* < 80 cases) (12, 13) as well as larger multicentric studies (14, 15) of thrombectomy showed that comparable outcomes could be achieved in clinical practice and randomized trials. In 2015–2016, >85% of patients with M1 occlusion admitted in our center were treated by thrombectomy. Functional independence (mRS 0-2) was obtained in 45% of these patients, which is similar to the proportion observed in the recent trials (46% in the Hermes meta-analysis) (17). Mortality was higher in our cohort than in this meta-analysis (20 and 15.3%, respectively), though it remained in the range of several controlled trials [18.4% (8) and 21% (4)]. Our exploratory analyses showed better outcomes for patients with M1 occlusion from 2015 onwards, indicating that our surge in EVT was clinically effective and safe. This improvement was most likely related to the ~3-fold increase in the proportion of patients with M1 occlusion who received EVT after our drastic change in acute treatment protocols (31.8 vs. 86.7% in 2013–2014 and 2015–2016, respectively). Nevertheless, we cannot rule out that some patients were selectively excluded from any reperfusion therapy (IVT or EVT) before or after 2015 (e.g., patients with very large ischemic core). As these patients were not included in our cohort, no matching procedure can account for this potential bias.

Higher reperfusion rates and shorter door-to-clot times were achieved after we implemented systematic EVT for patients deemed eligible. From 2015 onwards, both metrics (reperfusion rate: 71%; median door-to-clot time: 69 min) were on par with those found in randomized trials and currently suggested time benchmarks (e.g., door-to-puncture time of 75 min or less, imaging-to-puncture time of 50–60 min or less) (25). The substantial increase in EVT case volume likely contributed to these improvements. Previous data have consistently shown that high-volume centers achieved reperfusion faster and at higher rates, and with better clinical outcomes, than low-volume hospitals (26–28).

Our study has some limitations. Our data was not population-based, and therefore no exhaustive count of all ischemic stroke patients in our urban area was made. Thus, the true proportions of patients currently eligible for thrombectomy and of those who did receive this therapy, and any evolution of these ratios during the study period are not precisely known. We did not analyze the technical strategies used during EVT (e.g., maximum number of stent retriever passes, use of distal aspiration catheters); these may have evolved during the study period and influenced angiographic and clinical outcomes.

## Conclusion

Following the publication of the positive trials, thrombectomy was rapidly implemented with significant improvements in door-to-clot times and reperfusion rates. Accordingly, the outcome of patients with M1 occlusion also improved during the study period.

## Author contributions

LV and FF collected and analyzed the data, drafted and made critical revisions to the manuscript. JH analyzed the data and performed all statistical analyses, and made critical revisions to the manuscript. RR, LM, BG, EO, P-EL, LD, MH, LC, L-PB, RA, YB, and FT treated the patients and collected the data, and made critical revisions to the manuscript. NN and T-HC conceived the study, treated the patients, collected and analyzed the data, drafted and made critical revisions to the manuscript.

### Conflict of interest statement

The authors declare that the research was conducted in the absence of any commercial or financial relationships that could be construed as a potential conflict of interest.
